# Prospective validation of pediatric disease severity scores to predict mortality in Ugandan children presenting with malaria and non-malaria febrile illness

**DOI:** 10.1186/s13054-015-0773-4

**Published:** 2015-02-23

**Authors:** Andrea L Conroy, Michael Hawkes, Kyla Hayford, Sophie Namasopo, Robert O Opoka, Chandy C John, W Conrad Liles, Kevin C Kain

**Affiliations:** Depatment of Medicine, University of Toronto, Toronto, M5S1A8 Canada; Sandra A. Rotman Laboratories, Sandra Rotman Centre for Global Health, University Health Network-Toronto General Hospital, University of Toronto, Toronto, M5G1L7 Canada; Division of Pediatric Infectious Diseases, University of Alberta, Edmonton, T6G1C9 Canada; Department of Pediatrics, Jinja Regional Referral Hospital, P.O. Box 43 Jinja, Uganda; Department of Paediatrics and Child Health, Mulago Hospital and Makerere University, P.O. Box 7051, Kampala, Uganda; Division of Global Pediatrics, Department of Pediatrics, University of Minnesota, Minneapolis, MN 55414 USA; Department of Medicine, University of Washington, Seattle, WA 98195 USA; Tropical Disease Unit, Division of Infectious Diseases, Department of Medicine, University of Toronto, Toronto, Canada; Sandra Rotman Centre, Suite 10–351, Toronto Medical Discovery Tower, MaRS Centre, 101 College Street, Toronto, M5G1L7 Canada

## Abstract

**Introduction:**

The development of simple clinical tools to identify children at risk of death would enable rapid and rational implementation of lifesaving measures to reduce childhood mortality globally.

**Methods:**

We evaluated the ability of three clinical scoring systems to predict in-hospital mortality in a prospective observational study of Ugandan children with fever. We computed the Lambaréné Organ Dysfunction Score (LODS), Signs of Inflammation in Children that Kill (SICK), and the Pediatric Early Death Index for Africa (PEDIA). Model discrimination was evaluated by comparing areas under receiver operating characteristic curves (AUCs) and calibration was assessed using the Hosmer-Lemeshow goodness-of-fit test. Sub-analyses were performed in malaria versus non-malaria febrile illness (NMFI), and in early (≤48 hours) versus late (>48 hours) deaths.

**Results:**

In total, 2089 children with known outcomes were included in the study (99 deaths, 4.7% mortality). All three scoring systems yielded good discrimination (AUCs, 95% confidence interval (CI): LODS, 0.90, 0.88 to 0.91; SICK, 0.85, 0.83 to 0.86; PEDIA, 0.90, 0.88 to 0.91). Using the Youden index to identify the best cut-offs, LODS had the highest positive likelihood ratio (+LR, 95% CI: LODS, 6.5, 5.6 to 7.6; SICK, 4.4, 3.9 to 5.0; PEDIA, 4.4, 3.9 to 5.0), whereas PEDIA had the lowest negative likelihood ratio (−LR, 95% CI: LODS, 0.21, 0.1 to 0.3; SICK, 0.22, 0.1 to 0.3; PEDIA, 0.16, 0.1 to 0.3), LODS and PEDIA were well calibrated (*P* = 0.79 and *P* = 0.21 respectively), and had higher AUCs than SICK in discriminating between survivors and non-survivors in malaria (AUCs, 95% CI: LODS, 0.92, 0.90 to 0.93; SICK, 0.86, 0.84 to 0.87; PEDIA, 0.92, 0.90 to 0.93), but comparable AUCs in NMFI (AUCs, 95% CI: LODS, 0.86, 0.83 to 0.89; SICK, 0.82, 0.79 to 0.86; PEDIA, 0.87, 0.83 to 0.893). The majority of deaths in the study occurred early (n = 85, 85.9%) where LODS and PEDIA had good discrimination.

**Conclusions:**

All three scoring systems predicted outcome, but LODS holds the most promise as a clinical prognostic score based on its simplicity to compute, requirement for no equipment, and good discrimination.

**Electronic supplementary material:**

The online version of this article (doi:10.1186/s13054-015-0773-4) contains supplementary material, which is available to authorized users.

## Introduction

Since the implementation of Millennium Development Goal 4 to reduce childhood mortality, the number of deaths for children under five years of age has dropped from 11.9 million in 1990 to 6.3 million in 2013 [1,2]. Despite considerable success in reducing childhood mortality globally, it remains concentrated in the world’s poorest regions, with nearly half of under-five deaths in 2012 occurring in sub-Saharan Africa. Infectious diseases are important causes of death in children with pneumonia, malaria, measles, meningitis and HIV/AIDS accounting for over one-quarter of all under-five deaths in 2012 [3].

In-hospital deaths often occur within the first 48 hours of admission [4]. Implementation of simple and effective clinical tools to rapidly identify and treat the sickest children is urgently needed to reduce morbidity and mortality. Clinical scores can be used to assess disease severity in patients admitted to hospital, compare mortality rates between different institutions and regions, and evaluate the efficacy of different interventions to improve patient outcomes. A number of clinical severity scoring systems have been developed in pediatric populations, including PRISM (Pediatric Risk of Mortality Score) [5], PIM (Pediatric Index of Mortality) [6], sMODS (simplified Multi-Organ Dysfunction Score) [7], PELOD (paediatric logistic organ dysfunction) [8], PEWS (Pediatric Early Warning System Score) [9], bedside PEWS [10], and PAWS (Pediatric Advanced Warning System Score) [11]. However, many of these scores rely on laboratory data that are not available in many resource-constrained settings and are not practical for routine assessment of disease severity.

Recently, prognostic scoring systems have been specifically developed for pediatric populations in low-resource settings. These scores can be easily computed following patient assessment by front-line health care workers, without the need for specialized equipment, laboratory testing or onerous paperwork. Signs of Inflammation in Children that Kill (SICK) was developed in India as a practical triage tool based on data from 1,099 children (44 deaths) [12,13]. The Lambaréné Organ Dysfunction Score (LODS) was developed as a simple clinical prediction tool to identify African children with malaria in need of referral or close monitoring using data from 23,809 children (1,004 deaths) with severe *Plasmodium falciparum* malaria [14]. Finally, a score was developed for early death prediction in Kenyan children admitted to hospital (presented here as the Pediatric Early Death Index for Africa (PEDIA)) [15]. PEDIA was developed in a cohort of 8,091 children (436 deaths) admitted to hospital in Kenya and validated in a cohort of 4,802 children [15].

In this study we prospectively evaluated the ability of admission LODS, SICK and PEDIA to predict outcome in an observational study of febrile children admitted to hospital in Uganda. We assessed the discrimination and calibration of the scores and performed sub-group analysis to compare score performance in malaria versus non-malaria febrile illness (NMFI), and in early (≤48 hours) versus late (>48 hours) deaths.

## Methods

### Ethics

Ethical approval was granted from the Uganda National Council for Science and Technology, Makerere University Research Ethics Committee in Uganda, and the Toronto Academic Health Science Network. Written informed consent was provided by the accompanying parent or caregiver of all study participants.

### Study site and participants

This prospective observational cohort study was designed to evaluate predictors of mortality in febrile children presenting to Jinja Regional Referral Hospital in Uganda. The hospital serves a catchment area of three million people encompassing 12 districts in mid-eastern Uganda. The children’s unit has 100 beds, an average of 650 admissions per month and is manned by a clinical team that includes five pediatricians. The estimated entomological inoculation rate for Jinja is six infective bites per person per year, with malaria representing the most common admission diagnosis in the children’s unit [16].

The primary outcome for the study was all-cause in-hospital mortality. Inclusion criteria were: age two months to five years; parental report of fever within the past 48 hours, or axillary temperature greater than 37.5°C; hospitalization according to the admitting physician’s judgment; and parent/guardian consent to blood sampling and data collection. Outcome was assessed by daily chart review. Malaria was diagnosed using light microscopy of Field’s stained thick peripheral blood smear and three-band rapid diagnostic tests (RDTs) with *P. falciparum* histidine rich protein 2 (HRP2) and pan-malaria lactate dehydrogenase (pLDH) (First Response Malaria Ag. HRP2/pLDH Combo Rapid Diagnostic Test, Premier Medical Corporation Limited, India) [17]. Malaria was defined as any evidence of infection (direct visualization of parasites or detection of parasite antigen by either HRP2 or pLDH). Peripheral oxygen saturation (SpO_2_) was measured using the Masimo SET® Rad-57™ pulse co-oximeter (Masimo Corporation, Irvine, CA, USA).

### Clinical scores

The clinical scores were calculated according to the original methods and are briefly described [12,14,15].

#### Signs of Inflammation in Children that Kill (SICK)

SICK was calculated by adding up the pre-defined weightings for each abnormal variable in the score [12]. Level of consciousness was assessed using the AVPU score (‘Alert’, ‘Responding to Voice’, ‘Responding to Pain only’, or ‘Unresponsive’). Abnormal ranges and weightings for each variable were defined as: temperature >38°C or <36°C (weight, 1.2); heart rate >160/minutes (infant, <12 months of age) or >150/minute (child, ≥12 months of age) (weight 0.2); respiratory rate >60 (infant) or >50/minute (child) (weight, 0.4); systolic blood pressure <65 mm Hg (infant) or <75 (child) (weight, 1.2); SpO_2_ < 90% (weight, 1.4); capillary refill time ≥3 seconds (weight, 1.2); altered consciousness (unresponsive, responding to voice, or responding to pain only) (weight, 2.0). Additionally, weights were assigned based on the subject’s age (2 to <12 months, 1.0; ≥12 to <60 months, 0.3). Each variable was summed according to the weight generating possible scores ranging from zero to 8.6.

#### Lambaréné Organ Dysfunction Score (LODS)

The LOD score was calculated by assigning a value of one for each of the following signs present in a child to develop a score ranging from zero to three: prostration (unable to sit unsupported or inability of infants <6 months to breastfeed); coma (Blantyre Coma Score <3); or deep breathing.

#### Pediatric Early Death Index for Africa (PEDIA)

PEDIA was calculated by adding the pre-defined weightings for each indicator present to generate a score from zero to eight [15]. Kwashiorkor was not included on our standardized case report forms and was, therefore, not included in the final score (weight, 1.0 in the original model). The simplified score was generated using the following indicators and weights: jaundice (weight, 1.0); subcostal indrawing (weight, 1.0); prostration ± seizure (weight, 2.0); altered consciousness (with seizures, weight, 2.0; without seizures, weight, 3.0); wasting (weight, 1.0).

### Statistical analysis

Analyses were performed using GraphPad Prism v6, SPSS v20, and MedCalc v13 software. Continuous data were analyzed by the Mann–Whitney U test, and categorical data were analyzed by the Pearson Chi-Square or Fisher’s exact test, as appropriate. Score discrimination was assessed using non-parametric receiver operating characteristic (ROC) curves and comparing the areas under the curve (AUCs) using the method of Delong *et al*. [18]. Optimal cut-offs in clinical severity scores were determined using the Youden Index, which assigns equal weighting to model sensitivity and specificity (J = max(sensitivity + specificity-1)). Model calibration was assessed using the Hosmer-Lemeshow goodness-of-fit test comparing the observed versus expected probability of death within each risk group/decile derived from logistic regression models with mortality as the dependent variable and the model (SICK, LODS, PEDIA) as independent variables. The rate of missing data was ascertained for each variable and is presented in Additional file 1: Table S1. A sensitivity analysis was performed to evaluate the impact of missing data on score performance. To evaluate how scores performed under different conditions, we performed stratified analyses looking at the etiology of fever (malaria versus NMFI) and time to death (early versus late).

### Role of the funding source

The funders had no role in the study design; in the collection, analysis and interpretation of data; in the writing of the report; or in the decision to submit the paper for publication.

## Results

**Figure 1:**
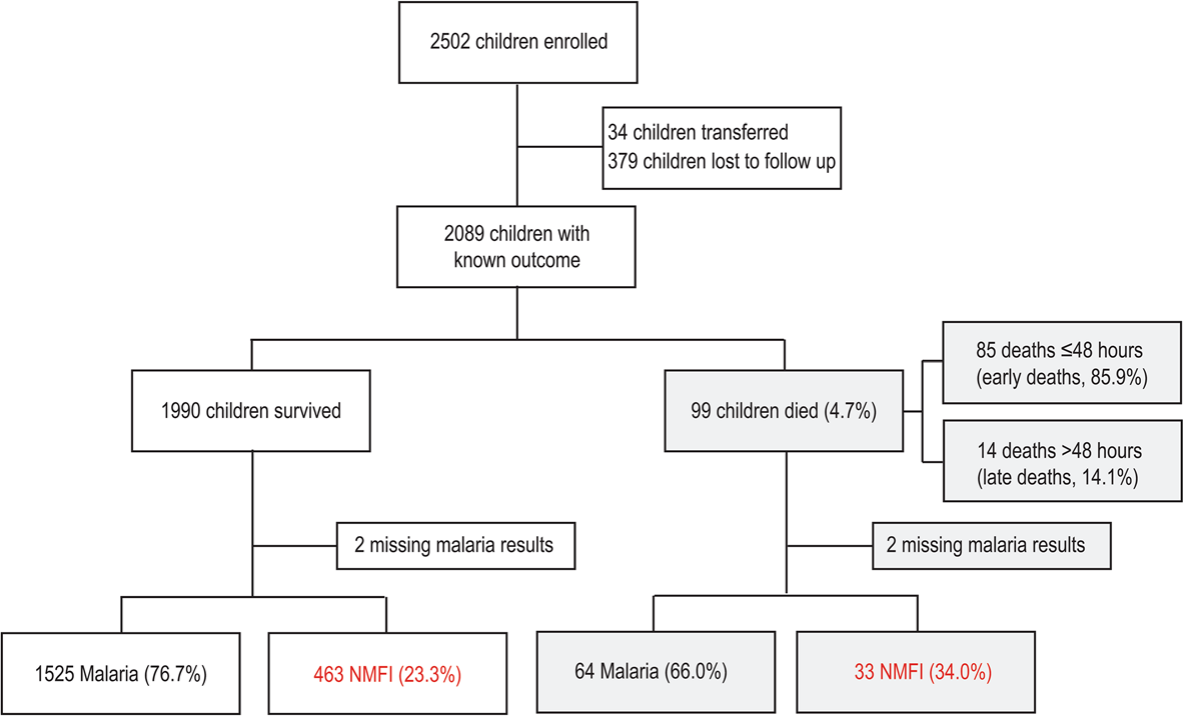
**Flow chart of children enrolled in the study.** A flow chart showing the children enrolled in the study and outcome stratified by malaria or non-malaria febrile illness (NMFI), or early (≤48 hours) versus late (>48 hours) study deaths.

A total of 2,502 children were recruited between February 2012 and August 2013 in a prospective observational cohort study to evaluate predictors of mortality among children hospitalized with fever (Figure 1). Outcome data were available for 2,089 children (1,990 survivors, 99 deaths) (Table 1). The all-cause mortality rate in children with known outcome was 4.7%. Among children with malaria, the mortality rate was 4.0% (64/1,525), whereas the mortality in NMFI was higher at 6.7% (33/463), *P* = 0.015. The mean age of children admitted to hospital was 20 months, and 54.3% were male. The mean time to see a medical doctor was 2.9 hours, and the mean duration of hospitalization was 3.1 days.

**Table 1:** **Demographic and clinical features**

**Variable**	**Survivors (number = 1990)**	**Non-survivors (number = 99)**	***P*** **-value**
**Demographic and anthropometric characteristics**			
Age — months	17.0 (9.0-26.5)	15.0 (9.0-25.0)	0.296
Age <12 months— number (%)	609 (30.7)	35 (35.4)	0.326
Male — number (%)	1079 (54.8)	55 (56.1)	0.797
Mid-upper-arm circumference — mm	140 (130–150)	130 (120–141)	<0.0001
Weight-for-age z < −3SD — number (%)	180 (9.3)	14 (14.7)	0.076
**Quality of care measures**			
Time to see medical doctor— hours	2.7 (1.0-4.3)	1.0 (0.2-2.8)	<0.0001
**Examination findings at admission**			
Axillary temperature—°C	38.0 (37.0-38.8)	37.4 (36.6-38.1)	<0.0001
Temperature >38°C— number (%)	891 (45.4)	26 (27.4)	0.001
Temperature <36°C — number (%)	70 (3.6)	11 (11.6)	<0.0001
Heart rate— beats/minute	162 (146–176)	163 (145–182)	0.839
Tachycardia— number (%)	1271 (64.4)	58 (63.0)	0.788
Respiratory rate— breaths/minute	42 (36–56)	60 (44–68)	<0.0001
Tachypnea— number (%)	490 (25.5)	50 (54.3)	<0.0001
Deep breathing— number (%)	434 (21.8)	77 (77.8)	<0.0001
Subcostal retractions— number (%)	353 (17.8)	66 (66.7)	<0.0001
Prostration— number (%)	440 (22.2)	85 (85.9)	<0.0001
Systolic blood pressure— mmHg	100 (100–110)	95 (90–103)	<0.0001
Hypotensive— number (%)	21 (1.1)	10 (12.3)	<0.0001
Capillary refill time ≥3 seconds — number (%)	86 (4.5)	20 (20.4)	<0.0001
Convulsions— number (%)	350 (17.6)	33 (33.3)	<0.0001
Altered consciousness— number (%)	282 (14.2)	76 (76.8)	<0.0001
Coma (BCS < 3) — number (%)	76 (3.9)	53 (53.5)	<0.0001
Jaundice— number (%)	188 (9.5)	25 (25.3)	<0.0001
**Laboratory test results at admission**			
Oxygen saturation	98.0 (96.0-99.0)	95.0 (88.0-99.0)	<0.0001
Oxygen saturation <90% — number (%)	62 (3.1)	25 (27.5)	<0.0001
Positive for malaria parasitemia — number (%)	1125 (63.0)	27 (40.3)	<0.0001
RDT Results — number (%)			
RDT negative	494 (25.6)	34 (35.8)	0.027
RDT pLDH positive	22 (1.1)	0 (0)	0.622
RDT HRP2 positive	237 (12.3)	19 (20.0)	0.037
RDT pLDH/HRP2 positive	1176 (61.0)	42 (44.2)	0.001
Glucose	6.9 (5.9-8.3)	5.4 (2.9-9.0)	<0.0001
Lactate	2.6 (1.9-4.6)	8.2 (2.9-16.9)	<0.0001
Hemoglobin	4.8 (3.4-7.0)	4.0 (2.4-6.7)	0.012
Positive for HIV antibody— number /total number (%)	39/931 (4.2)	6/37 (14.0)	0.003
**Duration of follow up — days**	3 (2–4)	1 (0–1)	<0.0001

Data presented as median (IQR). Tachycardia (heart rate >160/minute (infant, <12 months of age) or >150/minute (child, ≥12 months of age)); tachypnea (respiratory rate >60 (infant) or >50/minute (child)); hypotension (systolic blood pressure <65 mm Hg (infant) or <75 (child)). Prostration was defined as the inability to breastfeed or sit unsupported according to age. Altered Consciousness was defined using AVPU where anything except ‘alert’ constituted altered consciousness. BCS, Blantyre Coma Score; HRP2, *P. falciparum* histidine rich protein 2; pLDH, pan-malaria lactate dehydrogenase; RDT, rapid diagnostic test; SD, standard deviation.

### Model computation

**Figure 2:**
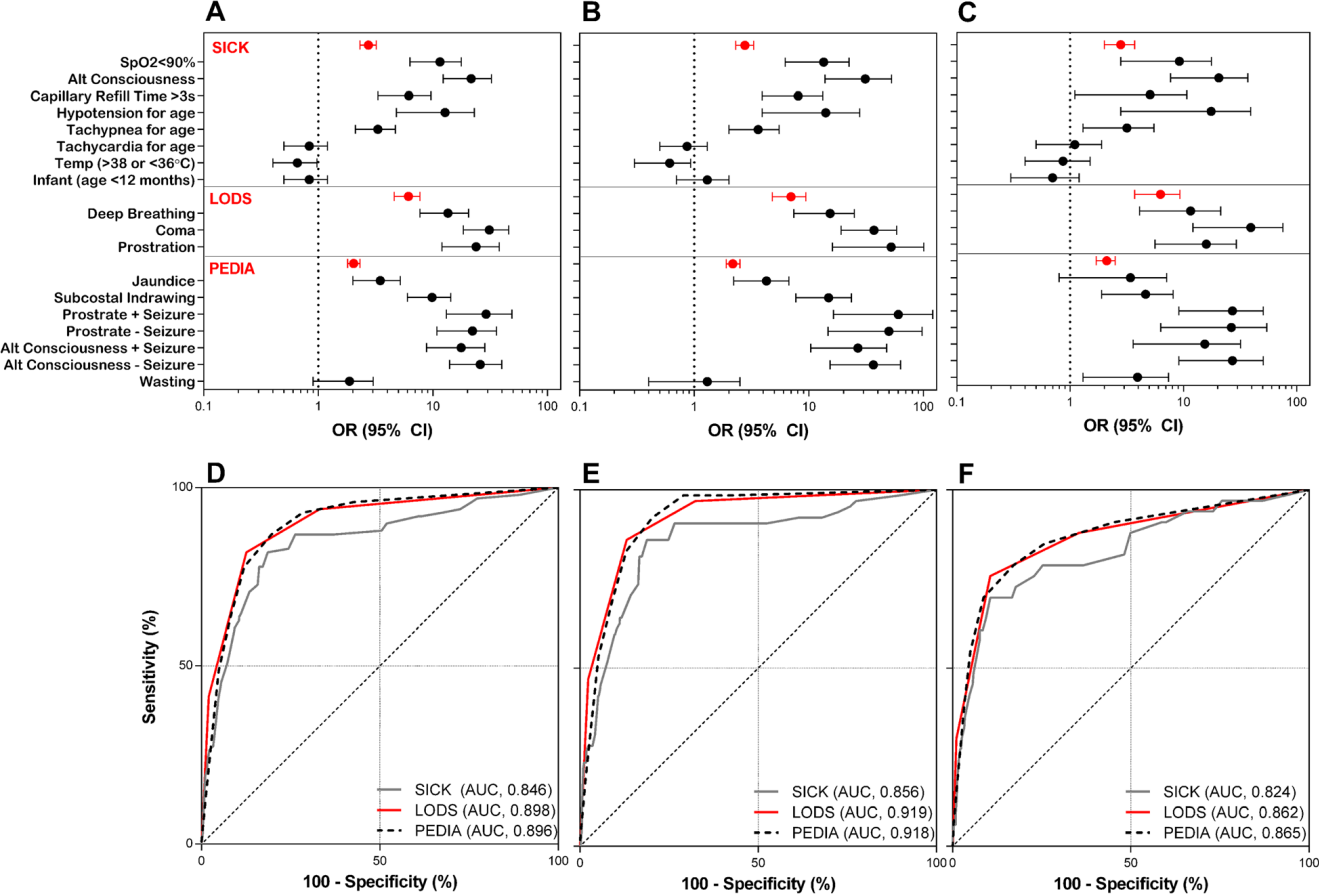
**Performance of SICK, LODS and PEDIA in discriminating between survivors and non-survivors.** Forest plots showing unadjusted odds ratios (95% CI) for the individual signs included in SICK, LODS, and PEDIA as well as the odds ratios of the clinical scores themselves (derived from logistic regression models) with mortality as the dependent variable in all children **(A** **)**, children with malaria **(B)**, and children with non-malaria febrile illness (NMFI, **C**). The associated receiver operating characteristic curves for A, B and C are shown in **D**, **E** and **F**. CI, confidence interval; LODS, Lambaréné Organ Dysfunction Score; PEDIA, Pediatric Early Death Index for Africa; SICK, Signs of Inflammation in Children that Kill.

When computing the disease severity scores, we first evaluated the association between individual variables included in the scores and mortality (Table 1). Forest plots were generated depicting unadjusted odds ratios for each clinical sign included in the scores, as well as for the scores themselves (derived from logistic regression models) (Figure 2, Additional file 1: Table S2). Among the variables included in SICK, younger age (<12 months versus 12 to 60 months), altered temperature and tachycardia were not associated with fatal outcome irrespective of the etiology of fever; in contrast, tachypnea, hypotension, altered capillary refill, altered consciousness and hypoxia were all associated with increased odds of death. Coma, deep breathing and prostration in LODS were associated with fatal outcome. In PEDIA, jaundice and subcostal indrawing were associated with fatal outcome, while wasting was of borderline significance (*P* = 0.076). Prostration and altered consciousness were considered in the context of whether a child had seizures. Seizures were associated with increased odds of death in prostration but not in children with impaired consciousness.

**Figure 3:**
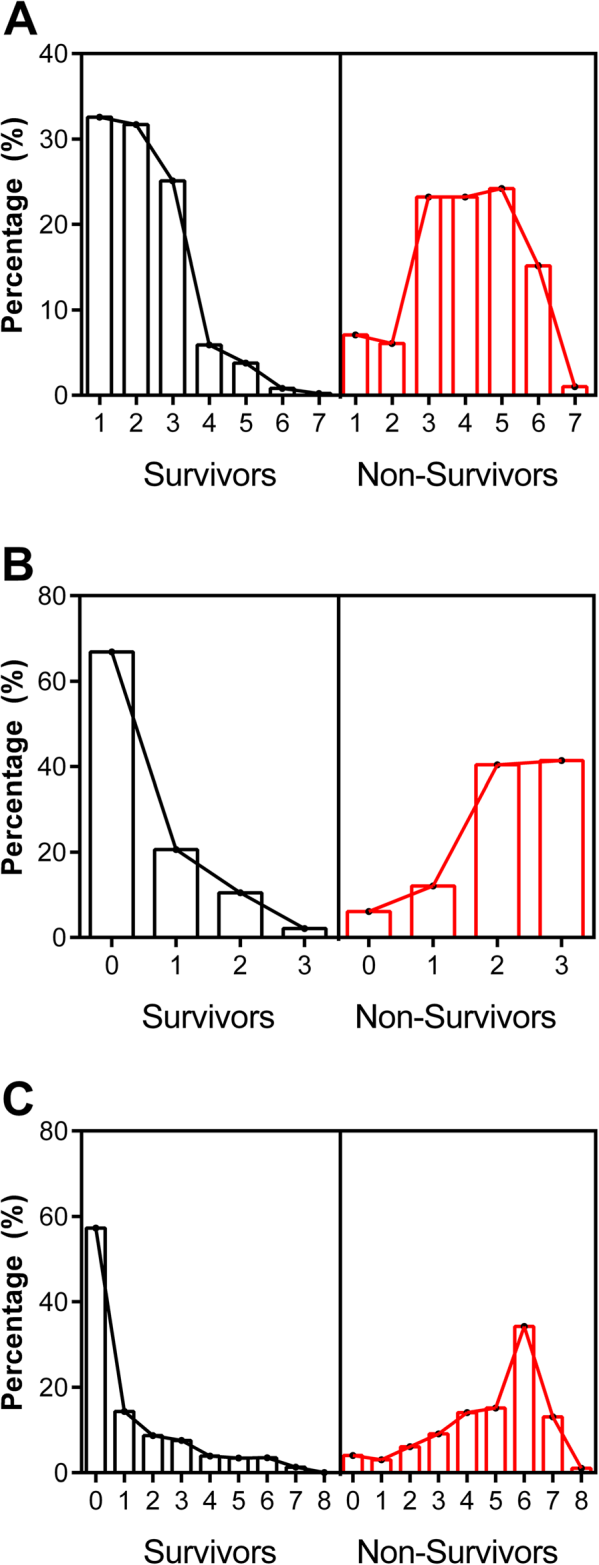
**The distribution of disease severity scores for SICK, LODS and PEDIA by mortality.** Histograms showing the distribution of disease severity scores for SICK **(A** **)**, LODS **(B)**, and PEDIA **(C** **)** as a percentage of survivors (left panel, in black) or non-survivors (right panel, in red). LODS, Lambaréné Organ Dysfunction Score; PEDIA, Pediatric Early Death Index for Africa; SICK, Signs of Inflammation in Children that Kill.

SICK scores ranged from 0 to 6.8 with a median score of 1.7 in survivors compared to 3.7 in non-survivors (*P* <0.0001, Mann–Whitney U test), with 86.9% of non-survivors having SICK scores above the median. Sixty four percent of children had none of the three signs for the LOD score, while 20% had one sign, 12% had two signs and 4% had all three signs. Among fatalities, 93.9% of children had a LOD score >1 with most children having a score of 2 (n = 40, 40.4%) or 3 (n = 41, 41.4%). PEDIA scores ranged from 0 (n = 1,144, 54.8%) to 8 (n = 1) with a median score of 0 in survivors compared to 5 in non-survivors (*P* <0.0001, Mann–Whitney U test). The distribution of disease severity scores for survivors and non-survivors is shown in Figure 3.

### Model discrimination and calibration

We compared the ability of SICK, LODS and PEDIA to discriminate between survivors and non-survivors using ROC curves. All three scores had good discriminatory ability with AUCs of 0.85 (95% CI, 0.83 to 0.86), 0.90 (0.88 to 0.91) and 0.90 (0.88 to 0.91) for SICK, LODS and PEDIA, respectively (Table 2, Figure 2). The AUCs for LODS and PEDIA were significantly higher than that for SICK (*P* = 0.03 and *P* = 0.02, respectively). We assessed model calibration for the models using the Hosmer-Lemeshow Goodness-of-fit test, with *P* <0.05 indicating a departure from perfect fit (Table 2). LODS and PEDIA were well calibrated, whereas SICK showed poor calibration in this population.

**Table 2:** **External validation of SICK, LODS and PEDIA in febrile Ugandan children admitted to hospital**

**Score**	**Discrimination AUC, 95% CI**	**Compare AUCs**	**Youden Index J, Cut-off**	**Sensitivity, 95% CI** ^**a**^	**Specificity, 95% CI** ^**a**^	**Calibration Hosmer-Lemeshow**
**Overall Score Performance**						
SICK	0.846, 0.83-0.86	*P* = 0.032 (S v. L)*	0.6328, >2.4	81.8, 72.8-88.9	81.5, 79.7-83.1	χ^2^ = 21.11, df = 8, *P* = 0.007
LODS	0.898, 0.88-0.91	*P* = 0.865 (L v. P)	0.6926, >1	81.8, 72.8-88.9	87.4, 85.9-88.9	χ^2^ = 0.072, df = 1, *P* = 0.789
PEDIA	0.896, 0.88-0.91	*P* = 0.016 (P v. S)*	0.6727, >2	86.9, 78.6-97.1	80.4, 78.6-82.1	χ^2^ = 4.45, df = 3, *P* = 0.217
**Modified Score Performance** ^**b**^						
SICK	0.854, 0.84-0.87	*P* = 0.059 (S v. L)	0.6269, >2.4	85.9, 77.4-92.0	76.8, 74.9-78.7	χ^2^ = 20.42, df = 8, *P* = 0.009
LODS	0.897, 0.88-0.91	*P* = 0.801 (L v. P)	0.6915, >1	81.8, 72.8-88.9	87.3, 85.8-88.8	χ^2^ = 0.066, df = 1, *P* = 0.797
PEDIA	0.894, 0.88-0.91	*P* = 0.041 (P v. S)*	0.6703, >3	86.9, 78.6-92.8	78.4, 76.5-80.2	χ^2^ = 3.60, df = 4, *P* = 0.462
**Malaria** ^**c**^						
SICK	0.856, 0.84-0.87	*P* = 0.036 (S v. L)*	0.6718, >2.4	85.9, 75.0-93.4	81.3, 79.2-83.2	χ^2^ = 20.54, df = 8, *P* = 0.008
LODS	0.919, 0.90-0.93	*P* = 0.964 (L v. P)	0.7282, >1	85.9, 75.0-93.4	86.9, 85.1-88.5	χ^2^ = 0.59, df = 1, *P* = 0.447
PEDIA	0.918, 0.90-0.93	*P* = 0.018 (P v. S)*	0.7186, >2	92.2, 82.7-97.4	79.7, 77.6-81.7	χ^2^ = 8.14, df = 3, *P* = 0.043
**NMFI** ^**d**^						
SICK	0.824, 0.79-0.86	*P* = 0.390 (S v. L)	0.5955, >2.9	72.7, 54.5-86.7	82.3, 78.5-85.7	χ^2^ = 6.79, df = 8, *P* = 0.560
LODS	0.862, 0.83-0.89	*P* = 0.859 (L v. P)	0.6517, >1	75.8, 57.7-88.9	89.4, 86.3-92.1	χ^2^ = 1.37, df = 1, *P* = 0.242
PEDIA	0.865, 0.83-0.89	*P* = 0.234 (P v. S)	0.6173, >2	78.8, 61.1-91.0	82.9, 79.2-86.3	χ^2^ = 0.34, df = 3, *P* = 0.952
**Early Death** ^**e**^						
SICK	0.853, 0.84-0.87	*P* = 0.009 (S v. L)*	0.6499, >2.4	83.5, 73.9-90.7	81.5, 79.7-83.1	χ^2^ = 15.66, df = 8, *P* = 0.049
LODS	0.919, 0.91-0.93	*P* = 0.396 (L v. P)	0.7450, >1	87.1, 78.0-93.4	87.4, 85.9-88.9	χ^2^ = 0.33, df = 1, P = 0.565
PEDIA	0.910, 0.90-0.92	*P* = 0.012 (P v. S)*	0.6981, >2	89.4, 80.8-95.0	80.4, 78.6-82.1	χ^2^ = 5.83, df = 3, *P* = 0.120
**Late Death** ^**f**^						
SICK	0.803, 0.79-0.82	*P* = 0.646 (S v. L)	0.5943, >2.2	71.4, 41.9-91.6	81.5, 79.7-83.1	χ^2^ = 9.88, df = 8, *P* = 0.273
LODS	0.769, 0.75-0.79	*P* = 0.243 (L v. P)	0.4546, >0	50.0, 23.0-77.0	87.4, 85.9-88.9	χ^2^ = 0.34, df = 1, *P* = 0.560
PEDIA	0.815, 0.80-0.83	*P* = 0.833 (P v. S)	0.5942, >3	71.4, 41.9-91.6	88.0, 86.5-89.4	χ^2^ = 0.061, df = 3, *P* = 0.996

Total data missing: LODS, 1.2%; SICK, 10.3%; PEDIA, 4.8%), default models consider missing data normal. ^a^Sensitivity and specificity calculated based on the following cut-offs (LODS, >1; SICK, >2.4; PEDIA, >2); ^b^missing data considered abnormal in score calculation (see Additional file 1: Table S1 for description of missing data by variable); ^c^malaria positive: positive by RDT (pLDH or HRP2) or microscopy (n = 1,589, 64 deaths; prevalence, 4.03%); ^d^malaria negative: negative by RDT (pLDH and HRP2) and microscopy (n = 496, 33 deaths; prevalence, 6.65%); ^e^n = 2,075 (1,990 survivors and 85 deaths); ^f^n = 2,004 (1,990 survivors and 14 deaths).*Compare AUCs (SICK, S; LODS, L; PEDIA, P) by method of Delong *et al*. AUC, area under the curve; CI, confidence interval; LODS, Lambaréné Organ Dysfunction Score; PEDIA, Pediatric Early Death Index for Africa; SICK, Signs of Inflammation in Children that Kill.

### Sensitivity analysis: effect of predictor variables on score performance

Complete data were available for 89.7% of children to compute SICK, 98.8% to compute LODS and 95.2% for PEDIA. When comparing the distribution of missing data, all variables included in LODS and PEDIA were missing at random, while five out of seven variables in the SICK score were more likely to be missing in children who died (heart rate, respiratory rate, systolic blood pressure, temperature, SpO_2_) (Additional file 1: Table S1). To evaluate the impact of missing data, we compared model discrimination and calibration for SICK, LODS and PEDIA when missing data were considered normal and then considered abnormal (Table 2). LODS and PEDIA had comparable discrimination irrespective of how missing data were handled. When missing data were treated as abnormal in the SICK score, the AUC improved from 0.846 (0.83 to 0.86) in the original model to 0.854 (0.84 to 0.87) in the modified score. The modified SICK score had higher sensitivity (86% versus 82%) but lower specificity (77% versus 82%) than the original. The decision to treat missing data as normal or abnormal did not affect model calibration.

### Score performance in malaria and non-malaria febrile illness

As LODS was developed exclusively in malaria and SICK was developed in a non-malaria setting, we wanted to evaluate the performance of each score in both malaria and NMFI. In addition, despite being developed in a cohort that included malaria and NMFI, PEDIA was developed and validated without consideration of clinical diagnosis. We wanted to assess the performance of all three scores in both malaria and NMFI. Of the 2,089 children with known outcome, 1,589 (76.1%) tested positive for malaria. The AUCs for SICK, LODS and PEDIA in discriminating between survivors and non-survivors in malaria and NMFI are shown in Figure 2 and Table 2. SICK and PEDIA were not well calibrated in malaria but showed good calibration in NMFI whereas LODS was well calibrated in both malaria and NMFI. Using a more specific definition of malaria (positive by three band RDT) [17], we obtained similar results.

### Performance of scores in predicting early or late death in hospital

**Figure 4:**
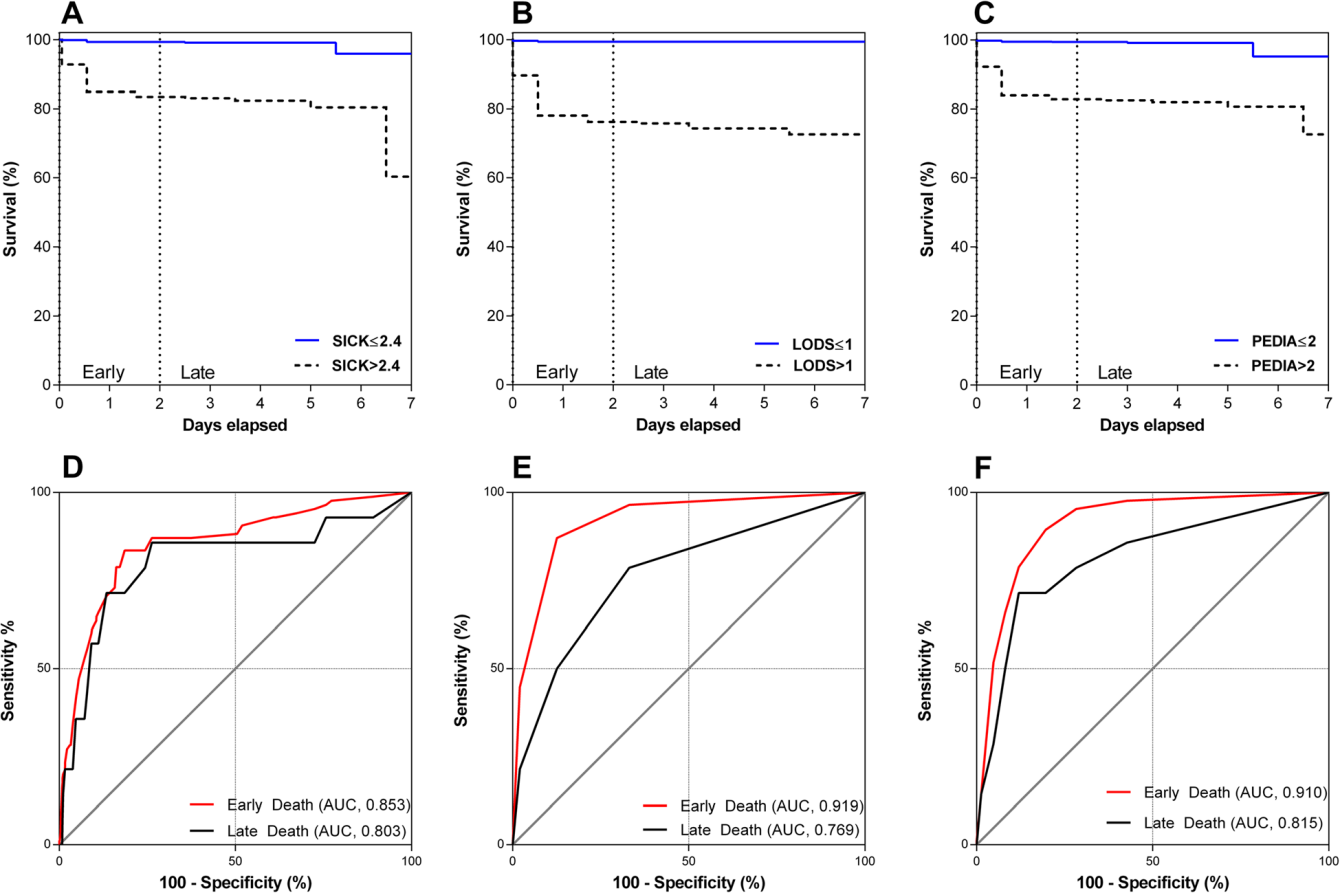
**Survival analysis and evaluation of SICK, LODS and PEDIA in predicting early versus late deaths in the first week of hospitalization.** Kaplan-Meier survival curves for each clinical score stratified according to the Youden index for SICK **(A** **)**, LODS **(B)** and PEDIA **(C)**. Receiver operating characteristic curves for SICK **(D)**, LODS **(E)**, and PEDIA **(F)** in discriminating between survivors and early (<48 hours) and late deaths (≥48 hours). LODS, Lambaréné Organ Dysfunction Score; PEDIA, Pediatric Early Death Index for Africa; SICK, Signs of Inflammation in Children that Kill.

Finally, we wanted to compare the scores in early and late mortality in our cohort. We plotted Kaplan-Meier survival curves with the clinical scores stratified according to their respective Youden indices. All scores were significantly associated with death (log-rank test, *P* <0.0001) (Figure 4), with the majority of deaths (n = 85, 85.9%) occurring in the first 48 hours of hospitalization (early deaths). SICK, LODS and PEDIA all had higher AUCs in early versus late deaths, but only LODS reached statistical significance (*P* = 0.036). LODS and PEDIA were better than SICK at discriminating between survivors and early deaths (*P* <0.05), but there were no differences between any of the AUCs for the clinical scores and late deaths (Table 2, Figure 4).

## Discussion

Clinical scoring systems are used in intensive care to compare performance between units and assess mortality in different patient groups. A similar system would be useful in resource-constrained settings where there is considerable heterogeneity in the quality of medical care. Clinical scores could be used to prioritize resources (at an individual patient level (that is, triage) and nationally/regionally), track changes in hospital performance over time, and monitor changes in disease patterns, in order to facilitate early identification of outbreaks. In this study, we validated SICK, LODS and PEDIA as prognostic scores for in-hospital mortality in a regional referral hospital in eastern Uganda. While all three scores were able to discriminate between survivors and non-survivors, LODS and PEDIA showed better discrimination and calibration in both malaria and NMFI. Of the three scores, LODS is the easiest to compute with only three variables compared to seven variables in SICK and eight variables in PEDIA, and had less missing data. As LODS had good discrimination and calibration, does not require equipment or specialized knowledge to generate, and is the most parsimonious, we believe it holds the most promise as a practical, ‘real world’ prognostic scoring system.

LODS was developed using the Severe Malaria in African Children (SMAC) network that collected data from six research sites (five countries) across Africa, including only children with laboratory-confirmed severe malaria [19]. Although the study validated the prognostic ability of LODS between disparate patient and parasite populations, and within different health care systems, it was not evaluated in NMFI. In this study, we present the first evaluation of LODS in the context of NMFI and show that each LODS sign (coma, prostration, deep breathing) was associated with a fatal outcome, irrespective of the etiology of fever. The performance of LODS declined significantly when trying to predict later deaths, which is consistent with recent SMAC reports showing considerable variability in model performance in predicting later deaths [20]. Although the predictors of early, intermediate and late death varied in subsequent analyses from the SMAC network, deep breathing, prostration and coma were significantly associated with intermediate and late deaths when data from all sites were analyzed together [20]. Differences in mortality kinetics and predictors of late mortality between sites may reflect regional differences in disease and quality of care. In our population, LODS had better sensitivity than the original cohort (94% versus 85%, LODS >0) and comparable specificity (98% versus 98%, LODS <3). Overall, LODS had good discrimination and calibration in malaria and NMFI, suggesting it may have widespread utility.

SICK was developed as a childhood triage score at a single tertiary care hospital in New Delhi [12,13]. The variables included in SICK were defined *a priori* based on physical manifestations of the systemic inflammatory response syndrome with weightings determined using multiple logistic regression analyses. In our study, not all variables included in SICK were associated with fatal outcome, suggesting a more parsimonious model could be developed. These findings are consistent with the original model where two variables (heart rate and respiratory rate) did not differ between surviving and non-surviving children, but were still included in the final model. The optimal cut-off in our study was >2.4 and was fairly consistent in sub-analyses, and similar to the cut-off derived from the development cohort (>2.5). Age was not associated with increased odds of death in our study; however, we only included children over 2 months of age. The neonatal period (first four weeks of life) carries one of the highest risks of death and accounts for over 40% of under-five deaths [21]. Had we included children of all ages in our cohort, age would likely have been an important predictor of death, as it was in the SICK development cohort. According to the developers of SICK, missing data should be treated as normal; however, in the context of our cohort we found better model discrimination if we considered missing data as abnormal since a number of variables were not missing at random. Despite these limitations, the discrimination of the modified SICK score was still less than LODS and PEDIA, suggesting that additional modifications to the SICK would be required before SICK should be considered as a practical scoring system in resource-constrained settings.

PEDIA is the only scoring system that was developed in Africa in children with both malaria and non-malaria illness [15]. In the original cohort used to develop the prognostic score, 56% of children admitted to hospital were positive for malaria. Thus, the score was developed in a large cohort of children with fever of mixed etiology. The original publication focused on developing prognostic models for immediate, early and late deaths. In this study, we elected to evaluate the early death score rather than the immediate score because it does not require any laboratory testing, whereas the immediate death score required assessment of anemia. PEDIA was comparable to LODS in predicting mortality among all children, children with malaria, NMFI, and those who died early (<48 hours) versus late. Generally, model calibration was also good. However, PEDIA is more complex than LODS without offering additional predictive/prognostic value, and does not provide additional clinical information that could be used to direct interventions (for example, fluids or oxygen).

To avoid subjective bias and explore consistency in score cut-offs under different conditions, we used a statistical method to define the optimal score cut-off for each analysis. We elected to use the Youden index, which gives equal weight to sensitivity and specificity. However, alternate methods could be used to establish cut-offs that would favor sensitivity or specificity. In the case of patient triage, increased sensitivity would be desirable. While the Youden index indicated a LOD score >1 had the best overall score performance in our cohort, selection of ≥1 as a cut-off would increase the sensitivity of the score from 81.8% to 93.9%. Likewise, the thresholds for SICK and PEDIA could be changed to reflect the desired sensitivity or specificity of the score. This will be an important consideration for future studies when assessing the score performance in different populations and disease conditions.

A limitation of this study was the inability to determine outcomes for all children, as approximately 15% of patients were lost to follow up. The reasons for the high rate of abscondment in the unit are unknown, but consistent with ongoing surveillance studies in the hospital. Clinically, the children who absconded were more likely to receive a blood transfusion (42.3%) than children who were discharged (33.7%) but less likely to receive anti-malarial treatment (quinine or artemisinin-based therapy). There were no differences in clinical diagnoses (malaria, pneumonia, sepsis and meningitis), pre-treatments or treatments (IV fluids, glucose, antibiotics) in hospital between children who were discharged and absconded. The absconders had higher LOD scores than survivors but lower LOD scores than non-survivors. In order to reliably assess patient outcomes in resource-constrained settings, it will be important to understand why patients abscond (for example, quality of care, direct/indirect costs, social reasons). Rates of missing data increased with increasing score complexity reaching 10.3% in the SICK score. While all variables in LODS and PEDIA were missing at random, several of the variables in SICK were more likely to be missing in children who died. When we evaluated which variables were more likely to be missing (Additional file 1: Table S1), it appears that variables requiring equipment (blood pressure, oxygen saturation, temperature) or direct assessment (heart rate, respiratory rate) had higher rates of missingness. These findings may reflect the limited resources of the site (in terms of both equipment and personnel), as well as the severity of illness, where documentation of vital signs was not prioritized in situations of critical illness.

Overall, 4.7% of the children in our study died, which is consistent with the mortality rates observed in the cohorts used to develop the LOD score (n = 23,890 children, 4.2% mortality) and PEDIA score (n = 8,091, 5.1% mortality). The sites used to develop these scores represent heterogeneous sites across Africa where malaria transmission intensity and the etiology of disease vary.

## Conclusions

This study is the first to compare clinical scores in a pediatric population in Africa including both malaria and NMFI. We demonstrated that LODS has good discrimination and calibration in NMFI, and evaluated SICK in a new population and disease context. Furthermore, we were able to validate PEDIA in a new population and demonstrate that it has good discrimination and calibration in febrile illnesses of different origin, and in early and late deaths. Overall, our data indicate that LODS is the most appropriate clinical prognostic score for resource-constrained settings based on its simplicity to compute, non-requirement for equipment, good discrimination and calibration, and suitability for implementation in both community and health care-based settings. Future studies should evaluate LODS in different malaria transmission settings and evaluate whether it can be integrated with readily available point-of-care test data (for example, hypoxemia) or host biomarkers of disease severity (for example, Ang-2) to improve risk prediction and/or guide therapeutics.

## Key messages

- New, simple and standardized clinical tools to objectively assess children at increased risk of death in resource constrained settings are urgently needed.
- Implementation of lifesaving measures to children at highest risk of adverse clinical outcomes in resource constrained settings can reduce child mortality.
- Of three clinical scoring systems developed to predict in-hospital mortality in developing world settings, the Lambaréné Organ Dysfunction Score shows the most promise as a simple, easy to compute score that predicts death in both malaria and non-malaria febrile illness.
- Implementation of disease severity scores in community and hospital-based settings could be an important tool to monitor progress in reducing child mortality and in evaluating new therapeutics.

## Additional file

Additional file 1: Table S1. Evaluation of missing data used in analysis in survivors and non-survivors. **Table S2.** Unadjusted odds ratios for fatal outcome based on clinical severity scores and individual variables.

## Abbreviations

AUC: area under the curve
BCS: Blantyre Coma Score
CI: confidence interval
HRP2: *P. falciparum* histidine rich protein 2
LODS: Lambaréné Organ Dysfunction Score
NMFI: non-malaria febrile illness
PEDIA: Pediatric Early Death Index for Africa
PELOD: pediatric logistic organ dysfunction
PIM: Pediatric Index of Mortality
pLDH: pan-malaria lactate dehydrogenase
PRISM: Pediatric Risk of Mortality Score
RDT: Rapid Diagnostic Test
ROC: receiver operating characteristic
SICK: Signs of Inflammation in Children that Kill
SMAC: Severe Malaria in African Children
sMODS: Simplified Multi-Organ Dysfunction Score
