# Supplementary material for: Prospective validation of pediatric disease severity scores to predict mortality in Ugandan children presenting with malaria and non-malaria febrile illness
Source: Crit Care. 2015 Feb 23;19(1):47. doi: 10.1186/s13054-015-0773-4 (PMC4339236; doi:10.1186/s13054-015-0773-4)
Supplement: Additional file 1: Table S1. — Evaluation of missing data used in analysis in survivors and non-survivors. Table S2. Unadjusted odds ratios for fatal outcome based on clinical severity scores and individual variables. [file 13054_2015_773_MOESM1_ESM.docx]

**Additional file**

**Table S1: Evaluation of missing data used in analysis in survivors and non-survivors**

|  | **Survivors (n=1990)** | **Non-Survivors**  **(n=99)** | **MAR**  **P-value** |
| --- | --- | --- | --- |
| **Demographic and anthropometric characteristics** |  |  |  |
| Age | 5 (0∙3) | 0 (0∙0) | 1∙000 |
| Wasting (weight-for-age z score <-3SD) | 48 (2∙4) | 0 (0∙0) | 1∙000 |
| **Examination findings at admission** |  |  |  |
| Axillary temperature | 26 (1∙3) | 4 (4∙0) | 0∙050 |
| Heart rate | 39 (2∙0) | 7 (7∙1) | 0∙001 |
| Respiratory rate | 66 (3∙3) | 7 (7∙1) | 0∙047 |
| Deep breathing | 3 (0∙2) | 0 (0∙0) | 1∙000 |
| Subcostal indrawing | 2 (0.1) | 0 (0∙0) | 1∙000 |
| Prostration | 4 (0∙2) | 0 (0∙0) | 1∙000 |
| Systolic blood pressure | 60 (3∙0) | 18 (18∙2) | <0∙001 |
| Capillary refill time | 64 (3∙2) | 1 (1∙0) | 0∙368 |
| Altered consciousness (AVPU) | 39 (2∙0) | 2 (2∙0) | 1∙000 |
| Coma (BCS<3) | 19 (1∙0) | 0 (0∙0) | 1∙000 |
| Convulsions | 4 (0∙0) | 0 (0∙0) | 1∙000 |
| Jaundice | 3 (0∙0) | 0 (0∙0) | 1∙000 |
| **Laboratory test results at admission** |  |  |  |
| Oxygen saturation | 19 (1∙0) | 8 (8∙1) | <0∙001 |
| Malaria microscopy | 203 (10∙2) | 32 (32∙3) | <0∙001 |
| RDT | 61 (3∙1) | 4 (4∙0) | 0∙547 |

No. (%) missing

**Supplementary Table S2: Unadjusted odds ratios for fatal outcome based on clinical severity scores and individual variables**

|  | **OR, 95% CI**  **All Children** | **OR, 95% CI**  **Malaria** | **OR, 95% CI**  **NMFI** |
| --- | --- | --- | --- |
| **SICK** | **2.7, 2.3-3.2** | **2.7, 2.3-3.3** | **2.7, 2.0-3.7** |
| Age <12 months | 0∙8, 0∙5-1∙2 | 1∙2, 0∙7-2∙0 | 0∙6, 0∙3-1∙2 |
| Abnormal temperature (>38 ˚C or < 36 ˚C) | 0∙6, 0∙4-0∙97 | 0∙6, 0∙3-0∙93 | 0∙7, 0∙4-1∙5 |
| Tachycardia | 0∙8, 0∙5-1∙2 | 0∙8, 0∙5-1∙3 | 0∙9, 0∙5-1∙9 |
| Tachypnea | 3∙1, 2∙1-4∙7 | 3∙3, 2∙0-5∙5 | 2∙7, 1∙3-5∙5 |
| Hypotensive | 10∙5, 4∙8-23∙0 | 10∙4, 3∙9-27∙8 | 10∙5, 2∙8-39∙3 |
| Capillary refill time ≥3 seconds | 5∙6, 3∙3-9∙6 | 7∙1, 3∙9-13∙2 | 3∙4, 1∙1-10∙7 |
| Altered consciousness | 20∙0, 12∙3-32∙4 | 26∙9, 13∙8-52∙2 | 16∙9, 7∙7-36∙9 |
| Oxygen saturation <90% | 10∙5, 6∙3-17∙7 | 11∙8, 6∙2-22∙4 | 7∙1, 2∙8-17∙7 |
| **LODS** | **6.0, 4.6-7.7** | **6.7, 4.8-9.4** | **5.8, 3.7-9.3** |
| Prostration | 21∙4, 12∙0-38∙0 | 40∙1, 16∙0-100∙6 | 12∙8, 5∙6-29∙3 |
| Coma (BCS<3) | 29∙0, 18∙4-45∙8 | 33∙4, 19∙1-58∙3 | 30∙3, 12∙1-75∙5 |
| Deep breathing | 12∙5, 7∙7-20∙4 | 13∙6, 7∙4-24∙9 | 9∙3, 4∙1-21∙3 |
| **PEDIA** | **2.0, 1.8-2.3** | **2.1, 1.9-2.5** | **2.1, 1.7-2.5** |
| Jaundice | 3.2, 2.0-5.2 | 3.9, 2.2-6.7 | 2.3, 0.8-7.1 |
| Subcostal indrawing | 9.2, 6.0-14.3 | 13.4, 7.7-23.5 | 3.9, 1.9-8.1 |
| Prostration with seizure | 25.3, 13.1-48.9 | 44.5, 16.4-120.2 | 18.6, 6.3-54.5 |
| Prostration without seizure | 19.7, 10.8-35.8 | 37.8, 14.7-97.1 | 11.1, 4.6-26.7 |
| Impaired consciousness with seizure | 15.8, 8.8-28.3 | 22.2, 10.4-47.6 | 10.8, 3.6-31.9 |
| Impaired consciousness without seizure | 23.6, 14.0-39.9 | 31.1, 15.3-63.0 | 21.5, 9.1-50.7 |
| Wasting (weight-for-age <-3SD) | 1.7, 0.9-3.0 | 1.0, 0.4-2.5 | 3.1, 1.3-7.4 |

Calculated from default models where missing data were considered normal
